# High-fidelity simulation is associated with good discriminability in emergency medicine residents’ in-training examinations

**DOI:** 10.1097/MD.0000000000026328

**Published:** 2021-06-18

**Authors:** Shou-Yen Chen, Chung-Hsien Chaou, Shiuan-Ruey Yu, Yu-Che Chang, Chip-Jin Ng, Pin Liu

**Affiliations:** a Department of Emergency Medicine, Chang Gung Memorial Hospital and Chang Gung University College of Medicine, Taoyuan; b Chang-Gung Medical Education Research Center; c Department of Emergency Medicine, West Garden Hospital, Taipei, Taiwan.

**Keywords:** emergency department, high-fidelity simulation, in-training examination, postgraduate medical education, resident training

## Abstract

In-training examinations (ITEs), arranged during residency training, evaluate the residents’ performances periodically. There is limited literature focusing on the effectiveness of resident ITEs in the format of simulation-based examinations, as compared to traditional oral or written tests. Our primary objective is to investigate the effectiveness and discriminative ability of high-fidelity simulation compared with other measurement formats in emergency medicine (EM) residency training program.

This is a retrospective cohort study. During the 5-year study period, 8 ITEs were administered to 68 EM residents, and 253 ITE measurements were collected. Different ITE scores were calculated and presented as mean and standard deviation. The ITEs were categorized into written, oral, or high-fidelity simulation test forms. Discrimination of ITE scores between different training years of residency was examined using a one-way analysis of variance test.

The high-fidelity simulation scores correlated to the progression of EM training, and residents in their fourth training year (R4) had the highest scores consistently, followed by R3, R2, and then R1. The oral test scores had similar results but not as consistent as the high-fidelity simulation tests. The written test scores distribution failed to discriminate the residents’ seniority. The high-fidelity simulation test had the best discriminative ability and better correlation between different EM residency training years comparing to other forms.

High-fidelity simulation tests had the good discriminative ability and were well correlated to the EM training year. We suggest high-fidelity simulation should be a part of ITE in training programs associated with critical or emergency patient cares.

## 1 Introduction

The goal of postgraduate medical education is to facilitate the resident's acquisition of medical knowledge and clinical skills and nurture the competency required to practice in a medical specialty.^[1]^ It is imperative to evaluate the residents’ performance periodically to help them overcome their weaknesses and ensure the quality of the residency program. The Accreditation Council for Graduate Medical Education (ACGME) and the Council of Residency Directors in Emergency Medicine emphasize the value of practical and reliable assessment tools to evaluate residents’ ability and the effectiveness of the residency programs.^[2–4]^

In-training examination (ITE), also known as in-service examination, was first introduced in 1963 to residents in the medical specialty by the American Academy of Orthopedic Surgeons.^[5]^ In either oral or written forms, ITE has been adopted by various medical specialties as a powerful and multi-functional assessment tool to measure the performance of residents.^[6–9]^ For EM residents, the ITE is not merely a signboard of their current academic performance but also a chance to prepare for the board exam. The ITE offers an opportunity for them to review their deficiencies in medical knowledge and to improve themselves.^[10]^ Prior studies have found a positive correlation between EM ITE scores and the American Board of Emergency Medicine written board certification scores.^[11–13]^ A similar correlation of the ITE and broad exam score was also documented in the field of internal medicine and its subspecialty of cardiology.^[14,15]^

Most of the ITEs and board-certification qualifying examinations usually consist of written and oral tests.^[6,13]^ Some of these types of examinations measure the degree of medical knowledge and clinical skills. However, they may not correlate to the residents’ overall clinical performance, which is supposed to advance with the residency training year. The use of oral and written tests is especially limited for programs conducted in a busy and rushing clinical environment, such as the emergency department (ED). The ED is notorious for the time limits, various independent tasks with undetermined priorities, and a rapidly changing environment full of interruptions.^[12]^ Written and oral test scores may not directly reflect the progress of clinical experience and multitasking ability by residency training year.

High-fidelity simulations, using computer-controlled mannequins, are being used throughout medical education to emulate real patient encounters since the 1960s.^[16,17]^ Besides, simulation is increasingly being used as an assessment tool to evaluate the performance of procedures.^[18,19]^ Simulation-based teaching was also integrated into the curriculum addressing the systems-based practice core competency and communication skill training course for residents.^[20–22]^ Simulation-based assessment can be used to evaluate the residents’ competency in differential diagnosis, resuscitation, and procedures, both formatively and summatively.^[23]^ Some literature supports the use of simulation-based assessment tools to evaluate residents, as in anesthesiology residency.^[24–26]^ Some even advocated for the use of simulation-based tests in board certification exams.^[19,27,28]^

There is currently limited literature focusing on the effectiveness of resident ITEs in the format of simulation-based examinations compared to traditional oral or written tests. Our primary objective is to compare the effectiveness and discriminative ability of high-fidelity simulation with written and oral tests in an EM training program.

## 2 Method

### 2.1 Study setting

This is a retrospective cohort study of EM resident physicians. The study was conducted at a university-affiliated tertiary teaching hospital with a 3600-bed capacity and an estimated annual ED volume of 180,000 patient visits. There are 63 board-certified EM faculty members within the department. The residency program accepts 7 to 10 resident physicians each year. The study was approved by our institutional review board (IRB no. 202000099B0).

### 2.2 Participants and data collection

In the EM program, ITEs were administered to all residents biannually, usually in February and August. In February, examinations usually involved 35 or 36 EM residents, and examinations in August had 26 to 29 residents each time. All residents needed to participate in the examinations unless specific conditions were met, such as severe illness. The first-year resident (R1) physicians would not take the examination in August because they usually registered in the same month. The ITEs were supervised by the residency program director and organized by the education committee within the department.

The ITE consists of 3 different forms: written examination, oral examination, and high-fidelity examination (oral and high-fidelity examination was canceled in 2018 due to equipment problems). The written examination involved multiple-choice questions (MCQs) and short answer question (SAQ) stations. MCQs had mixed content of EM. SAQs stations included electrocardiogram reading, image test (radiograph, computed tomography, and ultrasound), and other different EM themes. Oral tests included 2 to 3 stations with different EM topics, such as internal medicine, pediatrics, toxicology, emergency medical services, neurology, or disaster medicine. The rater of each station was an EM board-certified faculty member. A high-fidelity simulation station was operated with previously validated scenarios, a computer-controlled mannequin, 2 standardized nurses, and 1 faculty member as a rater. All of the simulation encounters were video recorded for post-test inspection.

The checklists of oral and simulation stations were validated before each ITE. The results of ITEs were regularly collected. Eight ITEs were included in the study. Only 1 ITE was administered in 2017 because of a change in the management level. Average scores from written, oral simulation, and high-fidelity exams were calculated. The written test, oral test, and high-fidelity examination scores were the mean scores across all stations.

### 2.3 Statistical analysis

Data were analyzed using SPSS software (version 13.0 for Windows; SPSS, Chicago, IL). In the descriptive analysis, categorical variables were presented as numbers and percentages. The reliability of ITE was evaluated using Cronbach's alpha coefficient. The discrimination of ITE scores between different training years of residency was examined using a one-way analysis of variance test. A *P* value <.05 was considered statistically significant.

## 3 Results

During the 5-year study period, 8 ITEs were administered to 68 EM residents training in our EM training program, and 253 ITE scores were collected. Table 1 reveals the characteristics of the ITEs and the participants. All 8 ITEs included written tests, and 7 of them contained oral tests and high-fidelity simulation tests.

**Table 1:** Characteristics of the ITEs and participants.

Characteristics of ITEs	Count
Total tests	8
Containing written test	8
Containing oral simulation test	7
Containing high-fidelity simulation test	7
Number of participating residents (lowest-highest)	26–36
Gender of participants	
Male	55
Female	13

Participants’ year of initiation	Male	Female	Total
2012	1	0	1
2013	8	1	9
2014	9	1	10
2015	8	2	10
2016	8	2	10
2017	7	3	10
2018	8	0	8
2019	6	4	10

The analysis of ITE content was revealed in Table 2. The oral test's most common topics were internal medicine and toxicology, whereas trauma and critical care most frequently appeared in the high-fidelity simulation tests. All the stations of SAQs, oral tests, and high-fidelity simulation tests were analyzed. The most discriminative subject is internal medicine (85.71%), followed by pediatrics (80%) and toxicology (60%) (Table 3). Other topics revealed diverse results. The internal consistency of the ITE was 0.95, which indicated good reliability across different participants.

**Table 2:** Characteristics of in-training exams.

Characteristics of ITEs	Count	(%)
Test number		
Total tests	8	
Written test	8	100
Oral simulation test	7	87.5
High-fidelity simulation test	7	87.5
Examination content		
Written test stations	38	
MCQs	7	18.42
SAQs stations	31	81.58
ECG	8	21.05
Image	6	15.79
Ultrasound	6	15.79
Critical care medicine	4	10.53
Gynecology	2	5.26
Others	5	13.16
Oral test stations	15	
Internal medicine	5	33.33
Pediatrics	3	20
Toxicology	4	26.67
Other	3	20
High-fidelity simulation stations	14	
Internal medicine	2	14.29
Trauma	6	42.86
Critical care	3	21.43
Pediatric	2	14.29
Toxicology	1	7.14
		

**Table 3:** Discriminability of in-training examinations, differentiated according to examination forms and test domains.

Discriminability according to different examination forms			
Examination	Written test	Oral simulation test	High-fidelity simulation
Test 1	*P* = .001^∗^	*P* < .001^∗^	*P* = .007^∗^
Test 2	*P* = .967	*P* = .730	*P* = .249
Test 3	*P* = .733	*P* = .001^∗^	*P* = .015^∗^
Test 4	*P* = .384	–	–
Test 5	*P* = .084	*P* = .006^∗^	*P* = .016^∗^
Test 6	*P* = .032	*P* < .001^∗^	*P* < .001^∗^
Test 7	*P* = .261	*P* = .001^∗^	*P* = .016^∗^
Test 8	*P* < .001^∗^	*P* < .001^∗^	*P* = .004^∗^

Discriminability according to test domains			
Test domain	Total stations	Discriminative stations	Discriminative prob (%)
Internal medicine	7	6	85.7
Pediatric	5	4	80.0
Toxicology	5	3	60.0
Mixed content (MCQ)	7	4	57.1
Critical care medicine	7	3	42.9
Trauma	7	3	42.9
Image	6	2	33.3
Sonography	6	1	16.7
ECG	8	1	12.5
Gynecology	2	0	(0)

**Figure 1:**
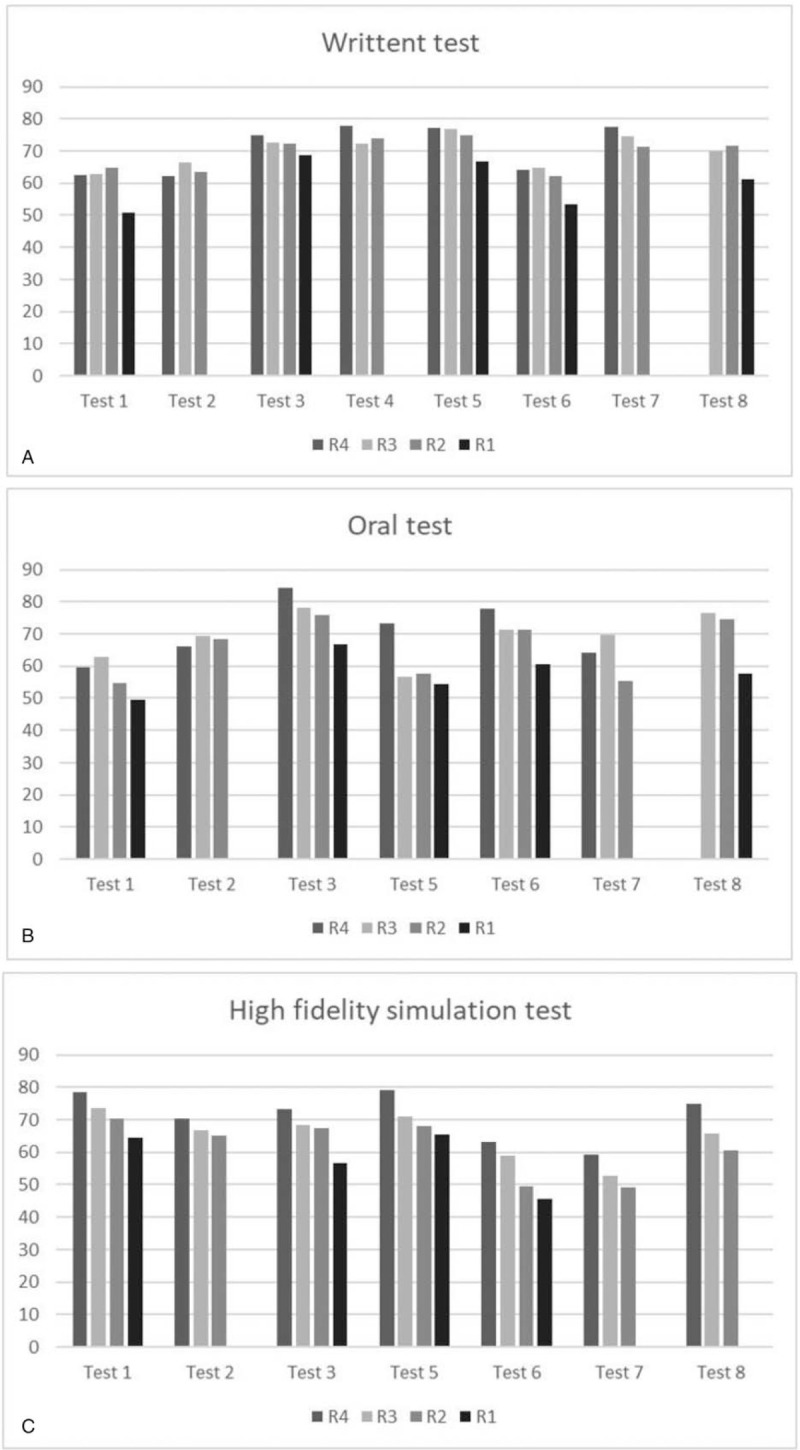
Illustration of test scores from (A) the written tests, (B) the oral tests, and (C) the high-fidelity simulation tests of residents in different training years.

Table 3 shows the discrimination of the tests according to the EM residency training year. Almost all oral and high-fidelity simulation tests of each exam were discriminative except one. The discrimination of the written tests was low. Although oral and high-fidelity simulation tests were both discriminative, the distribution of the scores of these 2 kinds of tests was different. Figure 1 demonstrates the average scores of different EM training year residents in each ITE. The high-fidelity simulation scores correlated to the EM training year, and R4 had the highest scores consistently, followed by R3, R2, and then R1 (Fig. 1C). The oral test scores (Fig. 1B) had similar results but not as consistent as the high-fidelity simulation tests. The written test scores distribution lacks similarity (Fig. 1B). The high-fidelity simulation test had the best discrimination and better correlation between different EM residency training years than the other 2 test forms.

## 4 Discussion

To the best of our knowledge, this is the first study to evaluate the utility of high-fidelity simulation in the ITEs of EM training programs. Our study demonstrated the good performance of a high-fidelity simulation test to discriminate the difference in EM residents’ competency in different training years. It may not be surprising that high-fidelity simulation tests had better results than written tests. As evaluated by written tests, medical knowledge can be obtained by increased reading, studying, and memorizing the core contents. Consequently, as long as he/she studies hard, a junior resident can outperform a senior one in a written test. Nonetheless, clinical experience obtained at the bedside gradually with EM residency training years cannot be rapidly obtained by goal-directed curriculum reading or short-term memory. A previous study also demonstrated improvement in written ITE scores after a structured board review program only in junior residents, but not senior ones.^[29]^ With the accumulation of “real-life” experience, medical knowledge should be integrated into the clinical scenario, including disease pattern recognition, applying the relevant algorithm, and immediate interventions to treat patients properly and timely.^[30]^ As a basis of competence in patient care, the clinical experience is much more than a simple memory recall. Rodgers’ study in advanced cardiac life support course concluded that written evaluation is a poor predictor of skill performance.^[31]^ Other studies also fail to use written and oral scores to predict residents’ clinical performance.^[6,13,30]^

Oral tests also showed good discrimination in our study. The junior EM residents (R1) almost always had inferior scores comparing to senior residents. Whereas the results were inconsistent in senior residents, and R2 or R3 physicians sometimes had better scores than R4 physicians in oral tests. One possible explanation is that oral tests may reflect both clinical experience and medical knowledge but not as realistic as high-fidelity simulation tests. Alternatively, high-fidelity simulation tests created a vivid scenario, which can evoke a physiologic response comparable to the real-life clinical situation.^[32]^ Therefore, high-fidelity simulation tests’ performance correlated better to the extent of clinical experience than oral tests. According to our result, assessment by high-fidelity simulation tests seems to be more appropriate to measure the EM residents’ clinical competency.

The high-fidelity simulation tests also had several advantages over traditional written and oral tests in assessing specific ACGME core competencies, including interpersonal and communication skills, professionalism, patient care, and systems-based practice.^[21,22]^ Medical educators’ direct evaluation through simulation-based assessment provides a simultaneous evaluation of knowledge, clinical reasoning, and teamwork.^[33]^ The standardization, fidelity, and reproducibility of medical simulation scenarios make it especially suited to be used in ITEs.^[18,23]^ With the advantages mentioned above, high-fidelity simulation is possibly a better assessment tool for EM training programs.^[34]^

ITE in EM training program provides the evaluation of medical knowledge and clinical competency and an opportunity for the residents to know their advantage and deficiency.^[10]^ Contrary to text-based learning and written tests, EM residents prefer question-based learning, which can be evaluated by simulation tests.^[10]^ Assessment by simulation had higher satisfaction rates when compared to written tests.^[35]^ Although high-fidelity simulation test is more expensive than a standard written or oral test, it may be more cost-effective than other commonly used assessment methods like “standardized patient” in Objective Structured Clinical Examination. Our study demonstrated the excellent discrimination of the high-fidelity simulation tests in the ITE of EM training programs. Although further study is needed, it should be considered as a part of the EM board certification examination.

### 4.1 Limitations

As demonstrated above, this is a single-center study. Local contexts needed to be taken into consideration before generalizing the study results. With its relatively small sample size, the statistical power of this study was may be limited. A larger confirmatory study under a different educational context should be performed in the future for a more conclusive result. In addition, this is a single-specialty study. We focused on the effectiveness of high-fidelity simulation tests in the ITEs of a high tension specialty as the EM. The results might not be fully applicable to other medical specialties with different characteristics of work. Finally, although all of the ITE raters were members of our education faculty with previous experiences in medical education and resident assessment, they did not receive additional training to improve their consensus and accuracy in evaluating the residents’ performances for each ITE.

## 5 Conclusion

High-fidelity simulation test used in ITE had a good discriminative ability and well correlated to the EM training year. We suggest high-fidelity simulation should be a part of ITE in training programs associated with critical or emergency patient cares.

## Author contributions

SYC contributed to the conceptualization, data collection and analysis, and draft writing of this research. CHC contributed to the research design, data acquisition, supervision of methodology, and data analysis. SRY contributed to the project administration, data acquisition, transcript coding, and participated in the analysis of themes. YCC contributed to the supervision of transcript coding and the emergence of themes. CJN contributed to the supervision of the project. PL contributed to the conceptualization of the research, software supervision, and qualitative data analysis. This is a unique submission and is not being considered for publication by any other source in any medium. All authors participated and contributed to the critical revision of the manuscript and gave final approval of the version submitted for publication.

**Conceptualization:** Shou-Yen Chen, Pin Liu.

**Data curation:** Shou-Yen Chen, Chung-Hsien Chaou, Shiuan-Ruey Yu.

**Formal analysis:** Shou-Yen Chen, Chung-Hsien Chaou, Pin Liu.

**Methodology:** Chung-Hsien Chaou.

**Project administration:** Shiuan-Ruey Yu.

**Software:** Pin Liu.

**Supervision:** Chung-Hsien Chaou, Yu-Che Chang, Chip-Jin Ng, Pin Liu.

**Writing – original draft:** Shou-Yen Chen.

**Writing – review & editing:** Pin Liu.
